# Multi-Channel Representation Learning Enhanced Unfolding Multi-Scale Compressed Sensing Network for High Quality Image Reconstruction

**DOI:** 10.3390/e25121579

**Published:** 2023-11-24

**Authors:** Chunyan Zeng, Shiyan Xia, Zhifeng Wang, Xiangkui Wan

**Affiliations:** 1 Hubei Key Laboratory for High-Efficiency Utilization of Solar Energy and Operation Control of Energy Storage System, Hubei University of Technology, Wuhan 430068, China; 2 Department of Digital Media Technology, Central China Normal University, Wuhan 430079, China

**Keywords:** compressed sensing, image reconstruction, deep unfolding network, attention mechanism

## Abstract

Deep Unfolding Networks (DUNs) serve as a predominant approach for Compressed Sensing (CS) reconstruction algorithms by harnessing optimization. However, a notable constraint within the DUN framework is the restriction to single-channel inputs and outputs at each stage during gradient descent computations. This constraint compels the feature maps of the proximal mapping module to undergo multi-channel to single-channel dimensionality reduction, resulting in limited feature characterization capabilities. Furthermore, most prevalent reconstruction networks rely on single-scale structures, neglecting the extraction of features from different scales, thereby impeding the overall reconstruction network’s performance. To address these limitations, this paper introduces a novel CS reconstruction network termed the Multi-channel and Multi-scale Unfolding Network (MMU-Net). MMU-Net embraces a multi-channel approach, featuring the incorporation of Adap-SKConv with an attention mechanism to facilitate the exchange of information between gradient terms and enhance the feature map’s characterization capacity. Moreover, a Multi-scale Block is introduced to extract multi-scale features, bolstering the network’s ability to characterize and reconstruct the images. Our study extensively evaluates MMU-Net’s performance across multiple benchmark datasets, including Urban100, Set11, BSD68, and the UC Merced Land Use Dataset, encompassing both natural and remote sensing images. The results of our study underscore the superior performance of MMU-Net in comparison to existing state-of-the-art CS methods.

## 1. Introduction

Compressed Sensing (CS) has revolutionized the limitations of the Nyquist sampling theorem, enabling the efficient reconstruction of signals at significantly lower sampling rates than the traditional Nyquist rate [1], particularly for signals exhibiting inherent sparsity or sparsity within specific transform domains [2]. This innovation has profound implications, substantially reducing the cost of sensor data compression, and mitigating the demands on transmission bandwidth and storage capacity in data transmission processes. CS has found wide applications, ranging from single-pixel cameras [3,4] to snapshot compression imaging [5,6] and even magnetic resonance imaging [7,8].

CS reconstruction methods can be broadly categorized into two main classes: traditional CS reconstruction methods [9,10,11,12,13,14,15,16] and deep-learning-based CS reconstruction methods [17,18,19,20,21]. Traditional CS reconstruction methods are designed based on a priori knowledge of image sparsity, presuming that the signal exhibits sparsity within a particular transform domain [22,23]. These methods formulate signal reconstruction as an optimization problem within a sparse model framework [12]. Solving this problem involves iterative approaches employing convex optimization methods, greedy algorithms, or Bayesian-like techniques to obtain the reconstructed signal. While traditional CS reconstruction methods provide strong convergence and theoretical guidance, they suffer from drawbacks such as computational intensity, slow reconstruction speeds, and limited reconstruction performance [24].

The computational complexity inherent in traditional CS reconstruction methods presents challenges in achieving real-time image reconstruction. To address this, deep learning methods, known for their prowess in image processing, have been introduced into the realm of CS reconstruction. Deep-learning-based CS reconstruction algorithms can be broadly classified into two primary categories: deep non-unfolding networks (DNUNs) [18,19,21,25,26] and deep unfolding networks (DUNs) [8,27,28,29,30,31,32,33]. DNUN treats the reconstruction process as a black-box operation, relying on a data-driven approach to build an end-to-end neural network to address the CS reconstruction problem. In this paradigm, the Gaussian random measurement matrix used in traditional CS reconstruction methods is replaced with a learnable measurement network. Subsequently, the reconstruction network framework is constructed around well-established deep learning models such as stacked denoising autoencoders [25], convolutional neural networks (CNNs) [18], or residual networks [26] to learn the mapping from CS measurements to reconstructed signals. Despite the ability of DNUN to achieve real-time reconstruction, surpassing traditional CS reconstruction methods, it has limitations such as high data dependency and poor interpretability, stemming from its entirely data-driven nature and lack of a strong theoretical foundation.

Conversely, DUN combines traditional optimization methods with deep learning techniques, utilizing optimization algorithms as theoretical guides. It employs a fixed-depth neural network to simulate the finite number of iterations of the optimization algorithm, resulting in reconstructed signals. Many optimization algorithms, such as Approximate Message Passing (AMP) [34], Iterative Shrinkage Thresholding Algorithm (ISTA) [35], and the Alternate Direction Multiplier Method (ADMM) [36], have been incorporated into DUN, leading to superior reconstruction performance compared to DNUN. Due to its foundation in theoretically guaranteed optimization algorithms, DUN offers strong reconstruction performance and a degree of interpretability.

Nonetheless, DUN typically operates in a single-channel form in many cases [27,28,29,30,37,38], as feature maps within the deep reconstruction network are transmitted between phases and updated within each phase. This structural characteristic limits the characterization ability of the feature maps, ultimately degrading the network’s reconstruction performance. Moreover, mainstream DUN methods [28,29,30,33,37,38] often rely on standard CNNs to build the reconstruction network, with each CNN featuring uniform receptive fields. As the human visual system is a multi-channel model, a series of receptive fields of different sizes are generated in the higher-order areas of the human visual system [39,40,41]. Therefore, the single receptive field of the standard CNN is inconsistent with the actual observation of the human visual system, which hampers the characterization ability of the CNN.

To address these limitations, this paper introduces two modules within the Deep Reconstruction Subnet (DRS) of our proposed Multi-channel and Multi-scale Unfolding Network (MMU-Net): the Attention-based Multi-channel Gradient Descent Module (AMGDM) and the Multi-scale Proximal Mapping Module (MPMM). These modules are designed to enhance feature characterization and representation in DUN. AMGDM facilitates the transmission of feature maps in a multi-channel format, both intra-stage and inter-stage. This design enhances the feature maps’ characterization ability. Moreover, inspired by SK-Net [42], we introduce Adap-SKConv, an attention convolution kernel with a feature fusion mechanism. Adap-SKConv is used to obtain fused gradient terms with attention, further improving the feature representation in AMGDM. To address the limitation of single-scale CNNs, we introduce MPMM, which employs multi-scale CNN. Inspired by the fact that the human visual system has different receptive fields in higher-order areas, in this paper, we utilize the Inception structure [43] and design Multi-scale Block (MB) with multiple parallel convolutional branches in MPMM to simulate the human visual system using different receptive fields to extract features, thus enhancing the network’s representational capability.

The main contributions of this paper are as follows:

- We introduce a novel end-to-end sampling and reconstruction network, named the Multi-channel and Multi-scale Unfolding Network (MMU-Net), comprising three integral components: the Sampling Subnet (SS), Initialize Subnet (IS), and Deep Reconstruction Subnet (DRS).
- Within the Deep Reconstruction Subnet (DRS), the Attention-based Multi-channel Gradient Descent Module (AMGDM) is developed. This module introduces a multi-channel strategy that effectively addresses the challenge of limited feature map characterization associated with the conventional single-channel approach. Additionally, we design the Adap-SKConv attention convolution kernel with a feature fusion mechanism, enhancing the feature characterization of gradient terms. These innovations collectively contribute to a substantial improvement in the network’s reconstruction performance.
- In DRS, we introduce the Multi-scale Proximal Mapping Module (MPMM). MPMM incorporates a Multi-scale Block (MB) featuring multiple parallel convolutional branches, facilitating the extraction of features across various receptive fields. This innovation allows for the acquisition of multi-scale features, significantly enhancing the characterization capabilities of the Convolutional Neural Network and thereby leading to an enhanced reconstruction performance.
- Empirical evidence from a multitude of experiments demonstrates the superior performance of the proposed method in comparison to existing state-of-the-art networks. This extensive validation underscores the efficacy and rationality of our approach.

The rest of the paper is organized as follows. Section 2 describes the related work of DNUN and DUN. Section 3 describes the preparatory knowledge for the work of this paper and Section 4 describes the framework and details of MMU-Net. Section 5 describes the experimental parameter settings, baseline, comparison with other state-of-the-art methods and ablation experiments. Section 6 draws the conclusions of the study.

## 2. Related Work

Deep-learning-based Compressed Sensing (DLCS) reconstruction networks can be categorized into two primary types: Deep Non-unfolding Networks and Deep Unfolding Networks. This section provides an exploration of the relevant work within each classification.

### 2.1. Deep Non-Unfolding Network (DNUN)

DNUN is characterized by its creation of end-to-end networks designed to execute the CS sampling and reconstruction processes. This approach leverages a data-driven strategy to acquire the knowledge necessary to map CS measurements into reconstructed signals. The initial foray into integrating deep learning into CS reconstruction was led by Mousavi et al. [25]. Their work employed stacked denoising autoencoders and feed-forward deep neural networks for signal reconstruction.

Subsequently, Kulkarni et al. [18] introduced ReconNet, which capitalized on fully connected layers and convolutional neural networks to reconstruct images. By substituting some of the fully connected layers with CNNs, ReconNet achieved superior performance, particularly in the realm of image processing. Yao et al. [26] presented DR2-Net, which initiated image reconstruction from CS measurements using fully connected layers. A residual network was then incorporated to further refine signal reconstruction.

Distinguishing itself from earlier CS reconstruction methods reliant on random Gaussian measurement matrix sampling, Shi et al. proposed CSNet [44]. This innovative approach harnessed CNNs to not only simulate the sampling process but also concurrently construct the sampling network, resulting in commendable reconstruction outcomes.

Building upon the foundation of CSNet, Shi et al. pursued several enhancements, introducing CSNet+ [45] and SCSNet [46]. These iterations further improved network reconstruction performance. However, DNUN’s significant drawback lies in its heavy reliance on data, inhibiting its versatility. Moreover, DNUN’s network structure is a product of a generic model, lacking theoretical grounding and interpretability due to deep learning’s inherent black-box nature, which can impede further optimization.

### 2.2. Deep Unfolding Network (DUN)

DUN represents a fusion of efficient deep learning models and optimization algorithms to construct deep reconstruction networks with pre-defined stages. Drawing inspiration from the Iterative Shrinkage Thresholding Algorithm, Zhang et al. introduced ISTA-Net and ISTA-Net+ [28]. These models unfolded each iteration into a network stage using CNNs, offering a promising balance between reconstruction performance and interpretability.

Zhang et al. further refined the concept with OPINE-Net+ [30], which replaced the random Gaussian measurement matrix with a learnable sampling matrix. This matrix incorporated orthogonal and binary constraints, while CNNs simulated the sampling and initial reconstruction processes, resulting in an adaptive end-to-end sampling and reconstruction network that notably improved reconstruction performance.

Building on the foundation of ISTA-Net+, You et al. introduced ISTA-Net++ [37]. This dynamic unfolding strategy addressed the challenge of CS sampling and reconstruction at varying sampling rates within a single model. The introduction of a cross-block strategy mitigated the chunking effect and further bolstered reconstruction performance.

Additionally, Zhang et al. conceived AMP-Net [29] based on the denoising perspective of the Approximate Message Passing algorithm. This model fashioned a sampling network through a random Gaussian matrix and crafted an unfolding network for deep reconstruction employing CNNs. This approach translated into highly efficient image reconstruction.

Song et al. addressed shortcomings in current DUN models related to short-term memory mechanisms. Their proposal, MAPUN [47], incorporated two distinct memory enhancement mechanisms, effectively reducing information loss between phases. This enhancement significantly improved the network’s expressive capacity and reconstruction performance.

***Summary:*** DUN surpasses both DNUN and traditional CS reconstruction methods in terms of reconstruction performance and interpretability. Consequently, it has become the prevailing approach in the field of CS reconstruction. Nevertheless, DUN is challenged by the need for multiple multi-channel to single-channel dimensional transformations during the reconstruction process, which can result in a loss of information and reduced feature map characterization capabilities. Additionally, the reliance on single-scale CNNs for reconstruction limits the network’s ability to extract image features from a single scale.

## 3. Preliminaries

This section provides a foundation for understanding the paper’s key concepts. It begins with a model of the Compressed Sensing task and subsequently introduces the Iterative Shrinkage Thresholding Algorithm and the Deep Unfolding Network framework based on ISTA. In this paper, vectors are represented using lowercase bold letters, matrices with uppercase bold letters, and parameters with italics. The important mathematical symbols and descriptions in this paper are shown in Table 1:

### 3.1. Problem Definition

**Definition** **1** (Compressed sensing problem). *The CS task encompasses two core components: sampling and reconstruction. Mathematically, the process of CS sampling can be expressed as follows (Equation (1)):*

(1) Y=ΦX

*Here, $X\in R^{N}$ signifies the original signal, $Y\in R^{M}$ represents the measurement, $\Phi \in R^{M\times N}$ is the random measurement matrix, and r=M/N denotes the sampling rate.*

*The CS reconstruction problem can be viewed as an ill-posed inverse problem. Traditional CS reconstruction methods approach this by solving Equation (2):*

(2) $$\min_{X}\frac{1}{2}{∥\Phi X-Y∥}_{2}^{2}+\lambda \Psi \left(X\right)$$

*Here, $\frac{1}{2}{∥\Phi X-Y∥}_{2}^{2}$ represents a data fidelity term, $\Psi \left(X\right)$ serves as a regularization term, ensuring that the solution adheres to prior information about the image, and λ denotes a regularization parameter.*

### 3.2. Definitions and Concepts

**Definition** **2** (ISTA-based DUN framework). *ISTA, a class of gradient algorithms, provides a classical approach for solving linear inverse problems. It accomplishes this by iterating through the following two main steps:*

(3) $$Z^{\left(k\right)}=X^{\left(k-1\right)}-\rho^{\left(k\right)}\left(\Phi^{⊤}\left(\Phi X^{\left(k-1\right)}\right)-\Phi^{⊤}Y\right)$$

(4) $$X^{\left(k\right)}=\underset{X}{\arg\min}\frac{1}{2}{∥X-Z^{\left(k\right)}∥}_{2}^{2}-\lambda {∥\Psi (X)∥}_{1}$$

*In Equation (3), $\rho^{\left(k\right)}$ denotes the step size, k represents the number of iterations, and $\Phi^{⊤}\left(\Phi X^{\left(k-1\right)}\right)-\Phi^{⊤}Y$ is the gradient of the data fidelity term in Equation (2). Equation (3) demonstrates that $X^{\left(k-1\right)}$ is updated in the direction of the negative gradient of the data fidelity term to produce the instant reconstruction result $Z^{\left(k\right)}$. Equation (4) showcases that the reconstruction result of the kth stage seeks $X^{\left(k\right)}$, approximating it to $Z^{\left(k\right)}$. Equation (4) can be viewed as a specialized form of proximal mapping, which can be converted to:*

(5) $$X^{\left(k\right)}=\underset{X}{\arg\min}\frac{1}{2}{∥F\left(X\right)-F\left(Z^{\left(k\right)}\right)∥}_{2}^{2}+\theta {∥F\left(X\right)∥}_{1}$$

*Here, $F\left(•\right)$ is a nonlinear sparse transform, and ISTA employs a soft threshold function to solve Equation (5):*

(6) $$X^{\left(k\right)}=\tilde{F}\left(soft\left(F\left(Z^{\left(k\right)}\right),\theta^{\left(k\right)}\right)\right)$$

*In Equation (6), $\tilde{F}\left(•\right)$ represents the inverse transformation of $F\left(•\right)$, and $soft\left(•,\theta^{\left(k\right)}\right)$ denotes the soft threshold function.*

**Figure 1:**
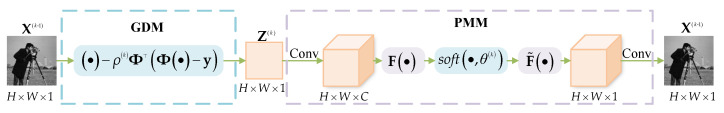
ISTA-based DUN network framework.

*The ISTA-based DUN network, based on Equations (3) and (6), establishes the network framework. The reconstruction network comprises Np stages, each encompassing a Gradient Descent Module (GDM) and Proximal Mapping Module (PMM), as depicted in Figure 1. The GDM corresponds to Equation (3) and simulates ISTA’s iterative step. It accepts the reconstructed image $X^{\left(k-1\right)}$ from the preceding stage as input and generates the instant reconstruction result $Z^{\left(k\right)}$ for the current stage. The GDM involves matrix operations on the feature maps without neural network participation, resulting in single-channel feature maps.*

*In the PMM, two nonlinear transformations, $F\left(•\right)$ and $\tilde{F}\left(•\right)$, designed based on Equation (6), typically consist of CNN modules. The input to PMM is a single-channel $Z^{\left(k\right)}$, initially converted into a multi-channel feature map through convolution. The multi-channel feature maps are then sequentially processed by $F\left(•\right)$, the soft thresholding function, and $\tilde{F}\left(•\right)$ to obtain a multi-channel feature map. Since GDM’s input is single-channel, and it operates solely on feature maps through matrix operations, feature maps remain single-channel throughout. However, PMM’s input and output are restricted to single channels. As a result, the module transforms input from multi-channel to single-channel, which results in information loss and constrains feature map characterization. Additionally, $F\left(•\right)$ and $\tilde{F}\left(•\right)$ are single-scale CNNs, which limits the network’s feature extraction capability.*

**Definition** **3** (CS ratio). *In this paper, $X\in R^{N}$ signifies the original signal, $Y\in R^{M}$ represents the measurement. The CS ratio is denoted by r, with r=M/N.*

**Definition** **4** (Multi-channel Representation Learning). *In this paper, “multi-channel” refers to the presence of multi-channel feature maps, meaning that the output of a network layer consists of feature maps with more than one channel. In contrast, “single-channel” feature maps have only one channel. Multi-channel feature maps can capture more diverse information than their single-channel counterparts.*

**Definition** **5** (Multi-scale CS Network). *The term “multi-scale” denotes the structure of a multi-scale network, which employs various convolutional kernels with different receptive fields, constructed in parallel to extract image features from different scales. This differs from a “single-scale” network that relies on a single type of convolutional kernel. Multi-scale networks can extract richer features.*

## 4. Proposed Method

**Figure 2:**
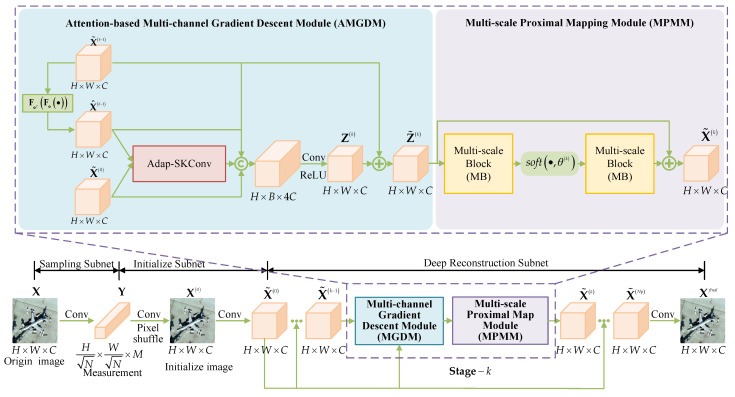
Network framework of the proposed MMU-Net.

In this section, we introduce the MMU-Net, which consists of three key sub-networks: the Sampling Subnet (SS), Initialize Subnet (IS), and Deep Reconstruction Subnet (DRS). The network’s architectural framework is illustrated in Figure 2, and the complete MMU-Net sampling and reconstruction process is detailed in Algorithm 1. The roles of these three sub-networks are as follows:

- **Sampling Subnet (SS)**: The SS emulates the linear sampling of the original image using convolutional layers. It transforms the input image to simulate the measurements obtained from a low-resolution sensor.
- **Initialize Subnet (IS)**: The IS operates on the measurements generated by SS. It enhances the dimension of these measurements to match the size of the original image and performs an initial reconstruction of the image.
- **Deep Reconstruction Subnet (DRS)**: The DRS unfolds the ISTA and progressively enhances the quality of image reconstruction over multiple stages. It refines the reconstruction in a stepwise manner, gradually approaching a higher fidelity output.

### 4.1. Sampling Subnet (SS)

In the Sampling Subnet, our approach assumes that the original image is represented as $X\in R^{H\times W}$. To process the image efficiently, it is divided into *L* blocks of size $\sqrt{N}\times \sqrt{N}$, where $\sqrt{N}\times \sqrt{N}\times L=H\times W$. This paper employs a layer of convolutional operations without biases, represented as $F_{\Phi }\left(•\right)$. Notably, we replace the traditional matrix sampling process with this convolutional layer. The sampling matrix Φ is treated as a learnable network parameter and reshaped into *M* convolutional kernels, each of size $\sqrt{N}\times \sqrt{N}$, with a step size of *N*. This process yields measurements Y with dimensions $\frac{H}{\sqrt{N}}\times \frac{W}{\sqrt{N}}\times W$, and it is mathematically expressed as:

(7) $$Y=F_{\Phi }\left(X\right)$$

### 4.2. Initialize Subnet (IS)

In the Initialize Subnet, the paper focuses on the initial reconstruction of the measurements Y into an image denoted as $X^{\left(0\right)}$. This process is facilitated by an unbiased convolutional layer $F_{\Phi^{⊤}}\left(•\right)$ and a Pixel Shuffle layer. The convolutional layer $F_{\Phi^{⊤}}\left(•\right)$ operates with a step size of 1 and employs *N* convolutional kernels of size 1×1×N, derived from the reshape of $\Phi^{⊤}$. In IS, the measurements Y first pass through $F_{\Phi^{⊤}}\left(•\right)$ to produce a feature map with dimensions $\frac{H}{\sqrt{N}}\times \frac{W}{\sqrt{N}}\times N$. Subsequently, the Pixel Shuffle layer reorganizes this feature map to generate the initial reconstruction image $X^{\left(0\right)}$ with dimensions H×W×1, as represented by the following equation:

(8) $$X^{\left(0\right)}=\mathrm{PixelShuffle}\left(F_{\Phi^{⊤}}\left(Y\right)\right)$$

**Algorithm 1:** Algorithm for constructing MMU-Net **Input**: Origin image X **Output**: reconstruction image $X^{final}$
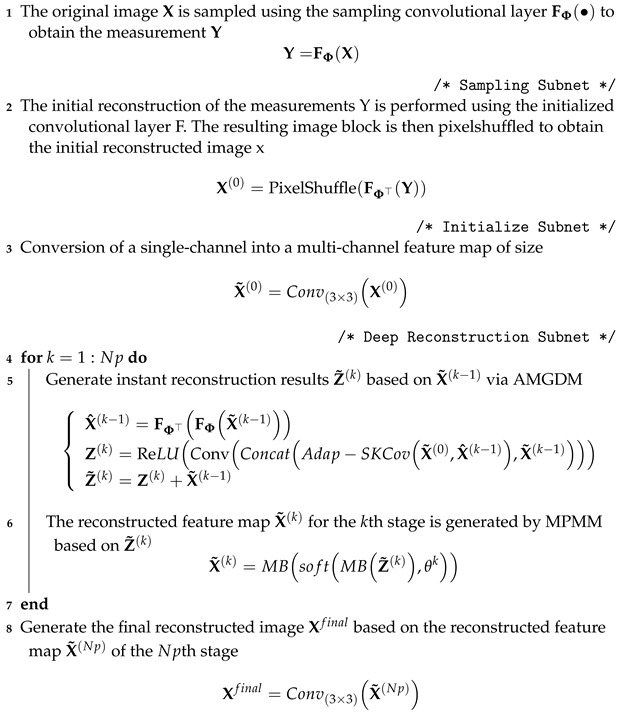
  Return: $X^{final}$


### 4.3. Deep Reconstruction Subnet (DRS)

The Deep Reconstruction Subnet in this paper employs Np stages by unfolding the ISTA. The DRS takes the initial image $X^{\left(0\right)}$ of size H×W×1 as its input. Initially, a 3 × 3 convolutional layer is used to transform the single-channel $X^{\left(0\right)}$ into a multi-channel feature map ${\tilde{X}}^{\left(0\right)}$ with dimensions H×W×C. Subsequently, based on the iterative updating steps of ISTA, the network is organized into Np stages, and each stage comprises two modules, namely, AMGDM and MPMM, corresponding to Equations (3) and (4). Finally, the multi-channel feature map ${\tilde{X}}^{\left(Np\right)}$ from the final stage is reduced to a single-channel image using a 3 × 3 convolutional layer, resulting in the final reconstructed image $X^{final}$.

To address the challenge of limited feature map characterization caused by the single-channel approach within DRS, a multi-channel strategy is incorporated into the AMGDM module. To ensure the rational allocation of weights among different channels, an Adap-SKConv approach with an attention mechanism is introduced to enhance the feature characterization of gradient terms in AMGDM. Additionally, to overcome the limitations of a single-scaled neural network with a restricted receptive field, the MPMM module employs multiple parallel convolutional branching structures (MB) to extract features across various receptive fields. This enables the capture of multi-scale features and enhances the network’s characterization capabilities.

#### 4.3.1. Attention-Based Multi-Channel Gradient Descent Module (AMGDM)

The structure of the AMGDM is designed based on Equation (3) in the iterative step of ISTA, and its position in the network framework is shown in Figure 2. AMGDM makes use of multi-channel versions ${\tilde{X}}^{\left(k-1\right)}$, ${\hat{X}}^{\left(k-1\right)}$, and ${\tilde{X}}^{\left(0\right)}$ of $X^{\left(k-1\right)}$, $\Phi^{⊤}\left(\Phi X^{\left(k-1\right)}\right)$, and $\Phi^{⊤}Y$ in Equation (3) to generate an instant reconstruction result $Z^{\left(k\right)}$. Notably, ${\hat{X}}^{\left(k-1\right)}$ is derived by applying $F_{\Phi^{⊤}}\left(F_{\Phi }\left(•\right)\right)$ channel-by-channel to ${\tilde{X}}^{\left(k-1\right)}$. The network framework is visually represented in Figure 2.

Specifically, the two gradient terms, ${\hat{X}}^{\left(k-1\right)}$ and ${\tilde{X}}^{\left(0\right)}$, are initially processed by the Adap-SKConv module to obtain a fused gradient feature map. Subsequently, this feature map is combined with ${\tilde{X}}^{\left(k-1\right)}$, ${\hat{X}}^{\left(k-1\right)}$, and ${\tilde{X}}^{\left(0\right)}$ to produce a feature map with dimensions H×W×4C. This feature map is then downscaled using a 3×3 convolutional layer followed by a ReLU activation function to yield an initial instant reconstruction result $Z^{\left(k\right)}$ of size H×W×C. Finally, ${\tilde{X}}^{\left(k-1\right)}$ is added to this result to obtain ${\tilde{Z}}^{\left(k\right)}$. The AMGDM operation can be represented as shown in Equation (9):

(9) X^k−1=FΦ⊤FΦX˜k−1Zk=ReLUConvConcatAdap−SKCovX˜0,X^k−1,X˜k−1Z˜k=Zk+X˜k−1

**Figure 3:**
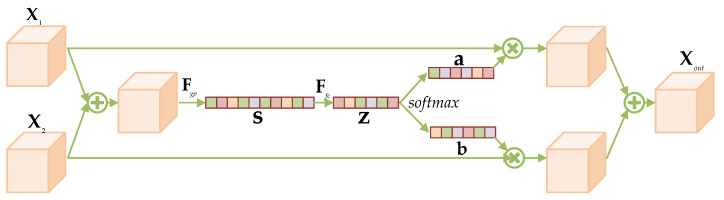
The network structure of Adap-SKConv.

In AMGDM, drawing inspiration from SKConv with multiple branches in SK-Net [42], Adap-SKConv incorporates an attention mechanism to fuse two feature inputs. The two gradient terms, ${\hat{X}}^{\left(k-1\right)}$ and ${\tilde{X}}^{\left(0\right)}$, are processed by Adap-SKConv to enhance the interaction between their information. This fusion enhances the feature characterization of gradient terms. The network structure of Adap-SKConv is visually depicted in Figure 3. Adap-SKConv accepts two inputs, $X_{1}$ and $X_{2}$. Initially, these inputs are fused, and global average pooling is performed to obtain global information on each channel, represented as the operation $F_{gp}$. This operation yields a vector s for each channel. Subsequently, a two-layered fully connected layer $F_{fc}$ is employed to obtain compact feature vectors z. Afterward, z undergoes softmax and segmentation to derive attentional weights a and b, corresponding to $X_{1}$ and $X_{2}$, respectively. Finally, $X_{1}$ and $X_{2}$ are multiplied and summed with a and b, respectively, to yield fused features $X_{out}$.

#### 4.3.2. Multi-Scale Proximal Mapping Module (MPMM)

The Multi-scale Proximal Mapping Module corresponds to Equation (6) and is responsible for solving proximal mapping through a soft threshold function and a nonlinear transformation. Its structure is depicted in Figure 2, and the operation can be expressed as shown in Equation (10):

(10) $${\tilde{X}}^{\left(k\right)}=MB\left(soft\left(MB\left({\tilde{Z}}^{\left(k\right)}\right),\theta^{\left(k\right)}\right)\right)$$

In this paper, the Multi-scale Block is employed to perform nonlinear transformations $F\left(•\right)$ and $\tilde{F}\left(•\right)$. MB leverages multiple parallel convolutional branching structures, inspired by Inception [43], to extract multi-scale features and enhance the characterization capabilities of the network. Notably, unlike classical ISTA-based Deep Unrolling Networks, the inputs and outputs of the Proximal Mapping Module in this paper are multi-channel feature maps rather than single-channel feature maps. Therefore, there is no need for a pre-$F\left(•\right)$ dimensional increase operation or a post-$\tilde{F}\left(•\right)$ dimensional reduction operation in MPMM to avoid information loss.

**Figure 4:**
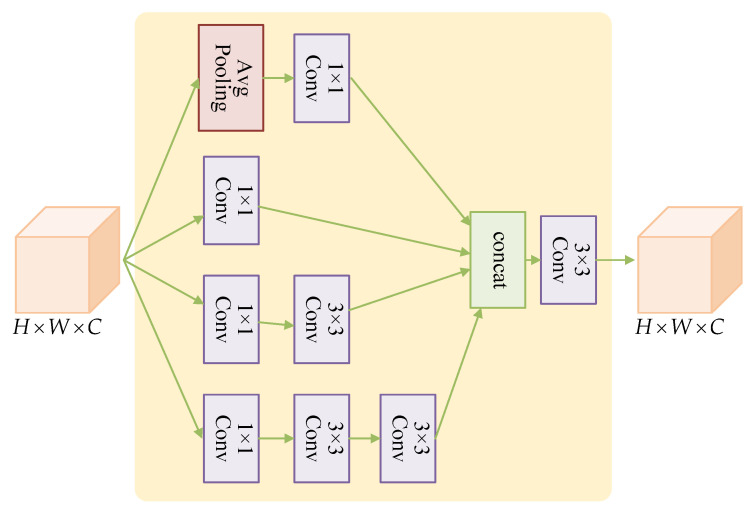
The network structure of Multi-scale Block.

The Multi-scale Block in MPMM adopts a parallel convolutional multi-branching structure inspired by Inception [43] to extract multi-scale features and enhance the network’s characterization abilities. The network structure of MB is visually presented in Figure 4, and the operation can be expressed as shown in Equation (11):

(11) $$\begin{array}{c} X^{out}=Conv_{\left(3\times 3\right)}\left(Concat\left(X^{b_{1}},X^{b_{2}},X^{b_{3}},X^{b_{4}}\right)\right)\\ with:\left\{\begin{array}{c} X^{b_{1}}=AvgPool\left(Conv_{\left(1\times 1\right)}\left(X^{in}\right)\right)\\ X^{b_{2}}=Conv_{\left(1\times 1\right)}\left(X^{in}\right)\\ X^{b_{3}}=Conv_{\left(1\times 1\right)}\left(\left(Conv_{\left(3\times 3\right)}\left(X^{in}\right)\right)\right)\\ X^{b_{4}}=Conv_{\left(1\times 1\right)}\left(Conv_{\left(3\times 3\right)}\left(\left(Conv_{\left(3\times 3\right)}\left(X^{in}\right)\right)\right)\right) \end{array}\right. \end{array}$$

The MB module is designed with four convolutional branches operating at different scales. The first branch includes a global average pooling layer and a convolutional layer with a kernel size of 1×1 and a ReLU activation function. The second branch consists of a convolutional layer with a kernel size of 1×1. The third branch comprises a convolutional layer with a kernel size of 1×1 and a convolutional layer with a kernel size of 3×3. The fourth branch consists of one convolution layer with a kernel size of 1×1 and two convolution layers with a kernel size of 3×3. The use of two 3×3 convolution kernels instead of 5×5 convolution kernels reduces the number of parameters while maintaining the same effective field and enhancing nonlinear representation. After feature extraction by these four branches from input features of size H×W×C, the resulting feature maps from the four different scales are concatenated. Finally, a convolutional layer group with a 3×3 kernel size is used for dimensionality reduction to yield an output feature map of size H×W×C. This results in multi-scale feature extraction and fusion.

### 4.4. Loss Function

The MMU-Net proposed in this paper comprises three sub-networks SS, IS, and DRS. During training, the network utilizes a dataset denoted as ${\left\{X_{i}\right\}}_{i=1}^{N_{b}}$, consisting of $N_{b}$ images, each with a size of $\sqrt{N}\times \sqrt{N}$. The entire MMU-Net is designed to optimize the following end-to-end loss function:

(12) $$\begin{array}{c} L_{total}=L_{discrepancy}+\gamma L_{orth}\\ with:\left\{\begin{array}{c} L_{discrepancy}=\frac{1}{NN_{b}}\sum_{i=1}^{N_{b}}{∥X_{i}-X_{i}^{final}∥}_{2}^{2}\\ L_{orth}=\frac{1}{M^{2}}{∥\Phi \Phi^{⊤}-I∥}_{2}^{2} \end{array}\right. \end{array}$$

Here, $L_{discrepancy}$ quantifies the mean square error between the original image $X_{i}$ and the final reconstructed image $X^{final}$. On the other hand, $L_{orth}$ enforces an orthogonality constraint on the sampling matrix. This constraint ensures that the rows of the sampling matrix exhibit minimal correlation, thereby reducing redundancy between observations. In the equation, I represents the identity matrix. The training procedure is outlined in Algorithm 2, with the hyperparameter γ in Equation (12) set to 0.01.
**Algorithm 2:** Training process of the proposed MMU-Net
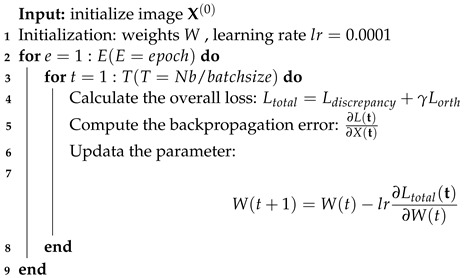


## 5. Experimental Results and Analysis

This section provides a comprehensive examination of the performance of our proposed MMU-Net. We begin by outlining our experimental settings, detailing the evaluation metrics used, and introducing the baseline methods. Subsequently, we delve into discussions that include an extended investigation, aiming to illustrate the efficacy of our method by addressing the following research questions:

***RQ1***: How does the performance of our proposed MMU-Net compare in accuracy to state-of-the-art CS reconstruction methods?

***RQ2***: What is the influence of the key components of the proposed AMGDM (including the multi-scale strategy and Adap-SKConv) in MMU-Net?

***RQ3***: What is the effect of the essential components (MB) of MPMM proposed in MMU-Net?

### 5.1. Experimental Parameter Settings

In our experiments, we employ a dataset comprising 91 images, consistent with previous work [30]. These images are utilized for training, with the luminance components of 88,912 randomly extracted image blocks, each of size 33×33, forming the training set. Our testing set encompasses three natural image datasets and a remote sensing image dataset. The nature image dataset consists of three widely recognized benchmark nature image datasets: Set11 [18], BSD100 [48], and Urban100 [49], and the remote sensing image dataset consists of eight images from the UC Merced Land Use Dataset [50].

For MMU-Net’s configuration, we set Np = 13, use a batch size of 32, establish a learning rate of $1\times 10^{-4}$, and run the training process for 300 epochs. During training, the network is optimized using an Adam optimizer [51] with a momentum of 0.9 and a weight decay of 0.999.

Our experiments are conducted using the Pytorch 1.11, and the hardware setup comprises an Intel Core i7-12700F processor and an RTX 3070 GPU. To evaluate the reconstruction quality, we utilize the Peak Signal to Noise Ratio (PSNR) and Structural Similarity Index Measure (SSIM) [52], focusing on the luminance components. In the results tables, the highest-performing method is indicated in bold, and the second-best is underlined.

### 5.2. Evaluation Metrics

#### 5.2.1. Peak Signal to Noise Ratio (PSNR)

PSNR is a widely-used metric for evaluating image quality at the pixel level. It measures the quality of a reconstructed image in decibels (dB), with higher values indicating superior image quality. For images X and Y, both of size m×n, the PSNR is computed as shown in Equation (13):

(13) $$\mathrm{PSNR}=10\cdot \log_{10}\left(\frac{{\mathrm{MAX}}_{X}^{2}}{\mathrm{MSE}}\right)$$

Here, ${\mathrm{MAX}}_{X}^{2}$ is the maximum possible pixel value of image X, and MSE denotes the mean square error between images X and Y.

#### 5.2.2. Structural Similarity Index Measure (SSIM)

SSIM is a metric that assesses image quality by quantifying structural similarity between two images. It provides insights into brightness, contrast, and structure, with SSIM values ranging from 0 to 1, where larger values indicate greater similarity between images. The SSIM between images X and Y is calculated according to Equation (14):

(14) $$\mathrm{SSIM}\left(X,Y\right)=\frac{\left(2\mu_{X}\mu_{Y}+c_{1}\right)\left(2\sigma_{\mathrm{XY}}+c_{2}\right)}{\left(\mu_{X}^{2}+\mu_{Y}^{2}+c_{1}\right)\left(\sigma_{X}^{2}+\sigma_{Y}^{2}+c_{2}\right)}$$

Here, $\mu_{X}$ and $\mu_{Y}$ represent the mean values of images X and Y, while $\sigma_{X}^{2}$ and $\sigma_{Y}^{2}$ represent their variances. The covariance between X and Y is denoted as $\sigma_{\mathrm{XY}}$. Additionally, $c_{1}$ and $c_{2}$ are constant terms.

### 5.3. Baselines

To gauge the effectiveness of MMU-Net, we conducted comparative evaluations by contrasting it with five well-established baseline methods. In this section, we provide an overview of these baseline techniques and their specific characteristics:

**AdapReconNet** [18]: AdapReconNet adopts a matrix sampling approach for chunked image sampling. It utilizes a fully connected layer for initial image reconstruction, while employing a variant of the ReconNet for deep reconstruction. Notably, the sampling matrix remains unaltered during the training phase, and the initial reconstruction subnetwork and deep reconstruction subnetwork are jointly trained.

**CSNet+** [45]: CSNet+ employs a convolutional neural network to accomplish chunked uniform sampling and chunked initial image reconstruction. Furthermore, it integrates a deep reconstruction sub-network. During the training phase, the sampling sub-network, initial reconstruction sub-network, and deep reconstruction sub-network are collectively trained.

**ISTA-Net+** [28]: ISTA-Net+ utilizes a fixed random Gaussian matrix for chunked image sampling and initial reconstruction. Deep image reconstruction is performed using an ISTA-based deep unfolding network. Similar to AdapReconNet, ISTA-Net+ maintains the sampling matrix constant throughout training and jointly trains the initial reconstruction and deep reconstruction sub-networks.

**OPINE-Net+** [30]: OPINE-Net+ integrates a CNN for chunked uniform sampling and chunked initial image reconstruction. It employs an ISTA-based deep unfolding network for the final image reconstruction. OPINE-Net+ extends the architecture of ISTA-Net+ by jointly training the look-alike network, the initial reconstruction sub-network, and the deep reconstruction sub-network.

**AMP-Net** [29]: AMP-Net initiates image reconstruction with a sampling matrix, initially set as a random Gaussian matrix. It performs chunked image sampling and initial reconstruction using this matrix. For the deep reconstruction phase, AMP-Net follows a denoising perspective, where a deep unfolding network is constructed based on the Approximate Message Passing algorithm. The sampling network, initial reconstruction sub-network, and deep reconstruction sub-network are collectively trained during the training phase.

### 5.4. Comparison with State-of-the-Art Methods (RQ1)

#### 5.4.1. Comparison in Natural Images

In this section, we compare MMU-Net with five state-of-the-art deep-learning-based CS reconstruction methods using four CS ratios: 0.04, 0.1, 0.25, and 0.3, under natural image datasets. The compared methods include AdapReconNet, CSNet+, ISTA-Net+, AMP-Net, and OPINE-Net+. AdapReconNet and CSNet+ belong to DNUNs, ISTA-Net+ and OPINE-Net+ are ISTA-based DUNs, and AMP-Net is an AMP-based DUN.

Table 2 presents the average PSNR and SSIM results of the five CS reconstruction methods on three datasets: Set11, BSDS68, and Urban100. The table illustrates that, across all four sampling rates, MMU-Net consistently outperforms the existing state-of-the-art CS reconstruction methods on Set11, BSDS68, and Urban100. This result confirms the efficacy of MMU-Net’s network structure. Notably, the DUN-based CS reconstruction methods demonstrate significantly better average PSNR and SSIM results compared to DNUN-based methods, suggesting the superiority of the DUN framework in enhancing reconstruction performance.

**Figure 5:**
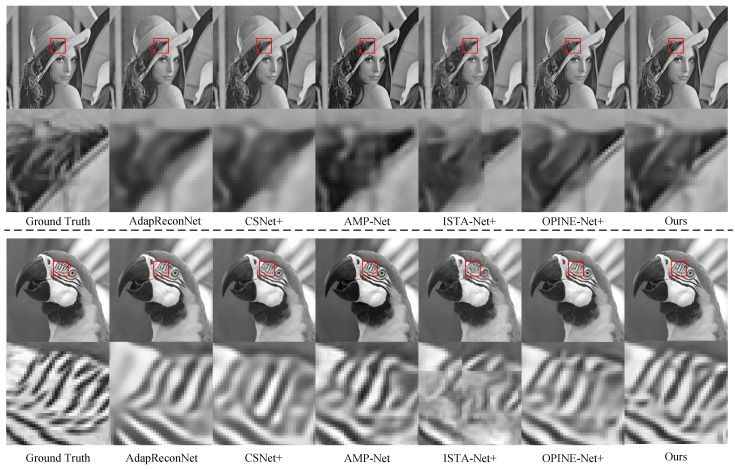
Reconstructed images generated by lena256 and Parrots in Set11 using six reconstruction methods at a sampling rate of 0.1, along with original images. Zoomed-in details are provided below each image.

Figure 5 displays the original images of lena256 and Parrots from the Set11 dataset, along with the images reconstructed by the seven CS reconstruction methods at a sampling rate of 0.1. The zoomed-in details of the reconstructed images are provided. The visual comparison reveals that the images reconstructed by MMU-Net exhibit minimal block artifacts and superior visual quality. A closer examination of the magnified image details of lena256 and Parrots underscores the richness of details and textures in the MMU-Net’s reconstructed images. In summary, MMU-Net outperforms the five state-of-the-art CS reconstruction methods in terms of average PSNR and SSIM while delivering superior visual quality.

#### 5.4.2. Comparison in Remote Sensing Images

**Figure 6:**
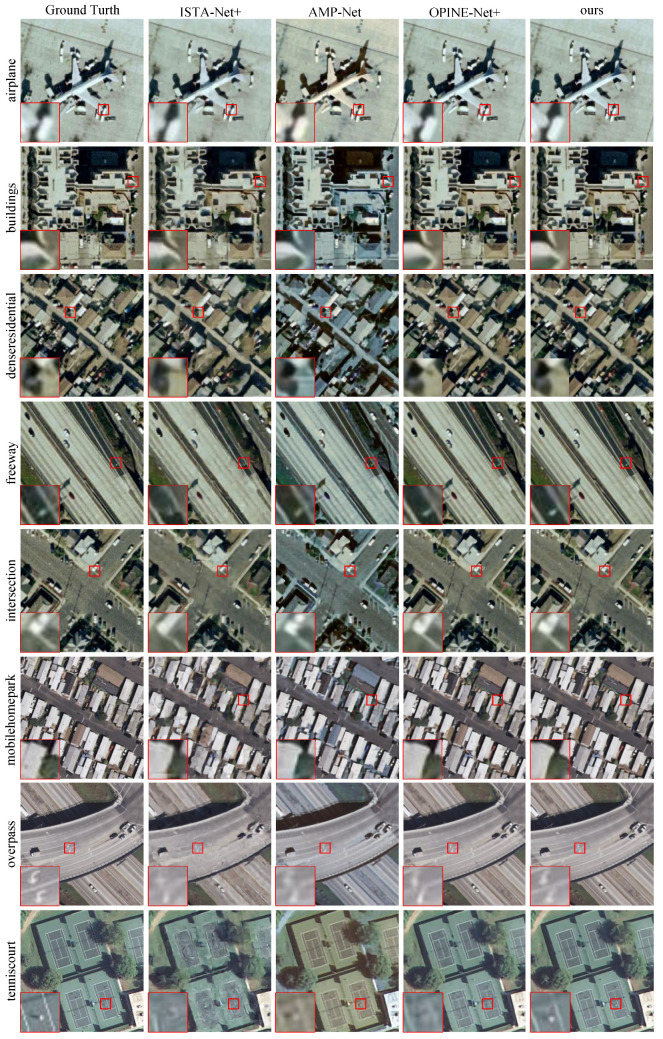
Eight different remote sensing images from the UC Merced Land Use Dataset are compared using the four methods at a sampling rate of 0.1. A zoomed-in view of the details is provided in the lower left corner of each image.

In this section, we assess the performance of MMU-Net using the UC Merced Land Use Dataset, a remote sensing image dataset. Based on our earlier findings favoring DUNs over DNUNs, we benchmark MMU-Net against three state-of-the-art DUNs: ISTA-Net+, AMP-Net, and OPINE-Net+. We evaluate the reconstruction quality at four different sampling rates: 0.04, 0.1, 0.25, and 0.3, with results visualized in Figure 6 and presented in Table 3.

The table showcases the average PSNR and SSIM values of reconstructed images for the four CS reconstruction methods across eight different remote sensing images. The results presented in Table 3 indicate that the PSNR of MMU-Net’s reconstructed images surpasses the second-best result by an average of 0.48 dB. Moreover, MMU-Net exhibits significantly better performance compared to the other three state-of-the-art CS reconstruction methods, underscoring the effectiveness of the MMU-Net’s network structure.

In Figure 6, we visually compare the reconstructed images and their corresponding originals at a sampling rate of 0.1 for various land-use classes. The lower-left corner of each image provides a magnified view of the selected area in the red box. As depicted in Figure 6, MMU-Net generates reconstructed images with clear contours and rich texture information. Importantly, it maintains the fidelity of small foreground targets even at lower sampling rates, ensuring that the target positions and shapes remain undistorted. In summary, the proposed MMU-Net excels in terms of both the average PSNR, SSIM, and visual quality, making it well-suited for demanding tasks such as target recognition in remote sensing images.

### 5.5. Study of Computational Time

In the context of CS reconstruction, the model’s reconstruction time and the number of parameters are crucial performance metrics. Typically, more complex network structures entail higher time complexity and a higher number of network parameters. In this section, two experiments are designed to validate the network performance of MMU-Net. The first compares the average GPU running time and the number of network parameters of MMU-Net with five other CS reconstruction algorithms. Comparison data are obtained by testing the same dataset in the same environment using the source code provided by the authors.The second explores the average GPU running time of MMU-Net on images of different sizes and the trend of the running time as the image size increases.

Table 4 provides the average GPU running times required by six CS reconstruction methods to reconstruct a 512 × 512 image at a sampling rate of 0.25. From the table, it is evident that the DNUN models, AdapReconNet and CSNet+, with relatively straightforward network architectures, exhibit shorter average running times in comparison to the DUN methods. In contrast, MMU-Net, the method proposed in this paper, has more expensive computation and preservation costs due to its multi-scale network structure and higher network complexity compared to other DUN methods. However, it still falls within the same order of magnitude as the other methods. Importantly, MMU-Net’s reconstruction performance surpasses that of the other methods.

**Figure 7:**
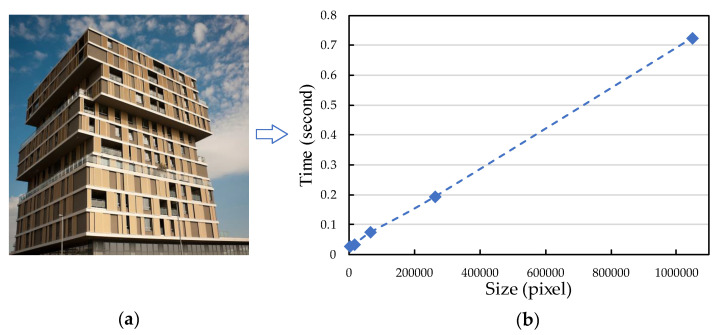
Visualization results of the average GPU runtime required to reconstruct the image on MMU-Net for five different sizes. (**a**) shows a building image in Urban100 of size 1024 × 1024, which is downsampled to obtain a series of images of 512 × 512, 256 × 256, 128 × 128 and 64 × 64. (**b**) shows a scatter plot of the average GPU runtime obtained by reconstructing the five image sizes on MMU-Net.

Figure 7 and Table 5 give the average GPU running time of MMU-Net, reconstructing images of sizes 64 × 64, 128 × 128, 256 × 256, 512 × 512 and 1024 × 1024, respectively. From the right panel of Figure 7, it can be seen that there is a near linear correlation between the average GPU running time of MMU-Net and the image size. When the input image size is large, the average GPU runtime of MMU-Net does not surge.

### 5.6. Ablation Studies and Discussions

In this section, we conduct ablation experiments to validate the effectiveness of the multi-channel strategy, Adap-SKConv, and the multi-scale strategy (MB).

#### 5.6.1. Effectiveness of AMGDM (RQ2)

To assess the effectiveness of the multi-channel strategy and Adap-SKConv within the AMGDM module, we utilize four network modules: GDM-(a), GDM-(b), GDM-(c), and GDM-(d), which replace the gradient descent modules at the locations shown in Figure 1. These modules allow us to compare network performance in different scenarios.

**Figure 8:**
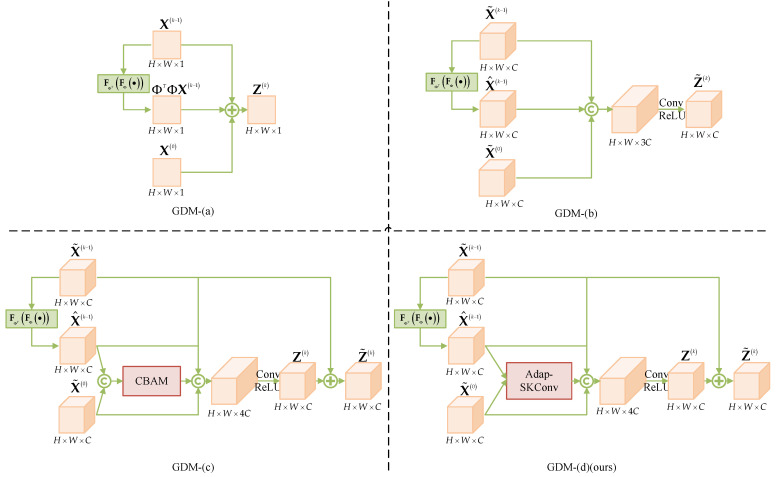
Network framework of GDM-(a), GDM-(b), GDM-(c) and GDM-(d).

GDM-(a) represents a single-channel module without an attention mechanism, similar to the GDM used in most ISTA-based DUNs. GDM-(b) is a multi-channel module without an attention mechanism. GDM-(c) incorporates a multi-channel module with the CBAM (Convolutional Block Attention Module) attention mechanism, which replaces the Adap-SKConv proposed in this paper. GDM-(d) is a multi-channel module with Adap-SKConv, i.e., the AMGDM proposed in this paper. The network structure of each module is illustrated in Figure 8.

GDM-(b), GDM-(c), and GDM-(d) all adopt multi-channel structures, thereby eliminating the need for subsequent PMMs to perform single-channel and multi-channel transformations, which reduces information loss. GDM-(c) and GDM-(d) utilize different attention mechanisms. Table 6 presents the average PSNR of these three methods on Set11 and the UC Merced Land Use Dataset at three different sampling rates.

From Table 6, we observe that the PSNR of the reconstructed images by GDM-(b) is, on average, 0.19 dB higher than that of GDM-(a) for the three sampling rates. This demonstrates that the multi-channel strategy proposed in this paper enhances the feature map characterization capability by mitigating the information loss resulting from dimensionality reduction, ultimately improving network performance. Additionally, when comparing GDM-(b) and GDM-(d), it is evident that the Adap-SKConv proposed in this paper contributes to an average gain of 0.17 dB in network performance. This confirms that Adap-SKConv effectively enhances the information exchange between gradient terms, thereby improving the quality of reconstruction through a well-designed attention mechanism. Lastly, when comparing GDM-(c) and GDM-(d) between Adap-SKConv proposed in this paper and the state-of-the-art CBAM attention mechanism, we find that the two-input structure of Adap-SKConv outperforms the single-input structure of CBAM in facilitating information exchange between the gradient terms. This enhances feature map characterization and, consequently, improves network reconstruction results.

#### 5.6.2. Effectiveness of MB (RQ3)

In this section, we conduct ablation experiments on the Multi-scale Blocks to assess the effectiveness of the multi-scale strategy, and the experimental results are included in Table 7.

**Figure 9:**
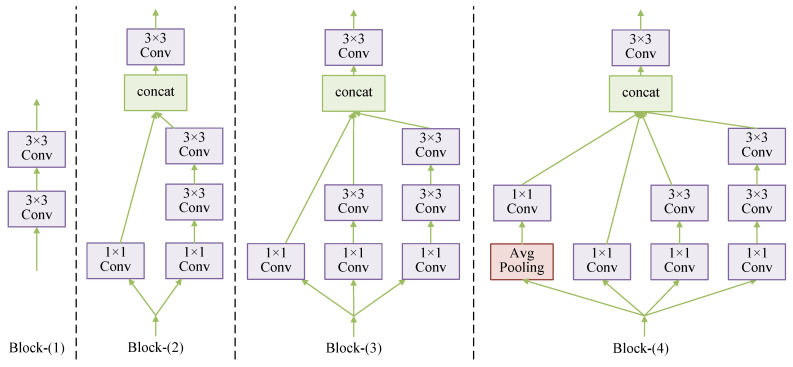
Network structure of Block-(1), Block-(2), Block-(3) and Block-(4).

We design and examine single-scale module Block-(1) and multi-scale modules Block-(2), Block-(3), and Block-(4), which comprise two, three, and four branches, respectively. Each of these modules is integrated into the network structure illustrated in Figure 1, replacing sections with $F\left(•\right)$ and $\tilde{F}\left(•\right)$. Among these modules, Block-(4) represents the MB designed in this paper. The structures of these four Blocks are visualized in Figure 9.

As shown in Table 7, the average Peak Signal-to-Noise Ratio of the reconstructed images increases with the number of branches. This observation confirms that the multi-scale strategy enhances network performance by increasing the network’s representation capability. However, as the number of branches increases, network complexity also rises, leading to longer training and reconstruction times. To strike a balance between performance and network complexity, this paper selects Block-(4) with four branches as the network structure for the proposed MB.

## 6. Conclusions

In this paper, we introduced a novel approach for Compressed Sensing image reconstruction. Our proposed MMU-Net leverages innovative strategies to enhance feature map characterization and gradient term representation, ultimately improving reconstruction performance. Specifically, MMU-Net incorporates a multi-channel strategy, bolstering the network’s ability to characterize feature maps effectively. In addition, the introduction of Adap-SKConv within the attention mechanism in Gradient Descent Modules facilitates the exchange of information between gradient terms, leading to improved representation capabilities. Furthermore, we introduced the Multi-scale Block, which enhances network characterization by introducing a multi-scale structure capable of extracting features at different scales. Our extensive experimental results demonstrate the superior performance of MMU-Net compared to state-of-the-art reconstruction algorithms. We have achieved a harmonious balance between algorithmic complexity and reconstruction quality, especially in the context of CS for natural and remote sensing images. The MMU-Net framework, as proposed in this paper, not only offers an effective solution for CS reconstruction in these domains but also opens up possibilities for enhancing a broad spectrum of applications, including image processing and computer vision. However, the MMU-Net proposed in this paper also has some limitations. First, due to the use of multi-channel and multi-scale strategy to build the network, resulting in more parameters in the model, the model requires further compression. Second, the method proposed in this paper adopts the block sampling strategy to improve sampling efficiency, and cannot realize the global pixel interaction, which limits the overall performance, and the feasibility of whole-map sampling needs to be further studied. For future research, we can direct our efforts toward further enhancing the performance of MMU-Net and exploring its applicability in diverse fields, promising continued advancements in image reconstruction techniques and their broader utility.

## Figures and Tables

**Table 1:** Mathematical notation and description.

Notations	Descriptions
*k*	Deep reconstruction sub-network stage index
X	Origin image, $X\in R^{N}$
Y	Measurement, $Y\in R^{M}$
*r*	CS ratio, r=M/N
$\Phi ,\Phi^{⊤}$	The sampling matrix, transpose of the sampling matrix
$F_{\Phi }\left(•\right),F_{\Phi^{⊤}}\left(•\right)$	Sampling convolutional layer, initialize convolution layer
$X^{\left(0\right)},X^{\left(k\right)}$	Initialize image, reconstruction image of the *k*th stage
${\tilde{X}}^{\left(k-1\right)},{\hat{X}}^{\left(k-1\right)},{\tilde{X}}^{\left(0\right)}$	The multi-channel versions of $X^{\left(k-1\right)}$, $\Phi^{⊤}\left(\Phi X^{\left(k-1\right)}\right)$, and $\Phi^{⊤}Y$
$Z^{\left(k\right)}$,${\tilde{Z}}^{\left(k\right)}$	The preliminary instant reconstruction result and the instant reconstruction result of the *k*th stage
$F_{gp}$,$F_{fc}$	The global average pooling, the two-layered fully connected layer
$\theta^{\left(k\right)}$	The threshold for the *k*th stage soft threshold function
$\rho^{\left(k\right)}$	The step size of the *k*th stage
$X^{final}$	Final reconstruction image

**Table 2:** Average PSNR and SSIM of reconstructed images for the six CS reconstruction methods across three datasets: Set11, BSDS68, and Urban100, and four sampling rates: 0.04, 0.1, 0.25, and 0.3. Bold indicates the best reconstruction performance, while underline represents the second-best reconstruction performance.

Dataset	Methods	CS Ratio			
		**0.04**	**0.1**	**0.25**	**0.3**
Set11	AdapReconNet [18]	23.87/0.7279	27.39/0.8521	31.75/0.9257	33.16/0.9379
	CSNet+ [45]	24.83/0.7480	28.34/0.8580	33.34/0.9387	34.30/0.9490
	ISTA-Net+ [28]	21.32/0.6037	26.64/0.8087	32.59/0.9254	33.74/0.9386
	AMP-Net [29]	24.64/0.7527	28.84/0.8765	34.42/0.9513	36.03/0.9586
	OPINE-Net+ [30]	25.65/0.7911	29.79/0.8905	34.81/0.9503	36.04/0.9600
	ours	**25.91/0.8008**	**30.17/0.8961**	**35.38/0.9555**	**36.62/0.9635**
BSDS68	AdapReconNet [18]	24.30/0.6491	26.72/0.7821	30.10/0.8901	30.54/0.9044
	CSNet+ [45]	25.43/0.6706	27.91/0.7938	31.12/0.9060	31.66/0.9152
	ISTA-Net+ [28]	22.17/0.5486	25.32/0.7022	29.36/0.8525	30.20/0.8771
	AMP-Net [29]	25.40/0.6534	27.79/0.7853	31.46/0.9053	32.84/0.9240
	OPINE-Net+ [30]	25.20/0.6818	27.72/0.8014	31.56/0.9121	32.50/0.9236
	ours	**25.29/0.6915**	**27.98/0.8097**	**31.76/0.9102**	**32.69/0.9259**
Urban100	AdapReconNet [18]	21.92/0.6390	24.55/0.7801	28.21/0.8841	29.71/0.9043
	CSNet+ [45]	21.96/0.6430	24.76/0.7899	28.13/0.8827	29.90/0.9162
	ISTA-Net+ [28]	19.83/0.5377	24.04/0.7378	29.78/0.8954	30.15/0.9070
	AMP-Net [29]	22.80/0.6814	26.04/0.8283	30.89/0.9202	32.19/0.9365
	OPINE-Net+ [30]	22.97/0.7018	26.51/0.8362	31.36/0.9216	32.58/0.9414
	ours	**23.35/0.7189**	**27.06/0.8474**	**31.96/0.9335**	**32.95/0.9442**

**Table 3:** Average PSNR and SSIM of the reconstructed images for the four CS reconstruction methods applied to the remote sensing image dataset at sampling rates of 0.04, 0.1, 0.25, and 0.3. Bold indicates the best reconstruction performance, while underline represents the second-best reconstruction performance.

Image	Methods	CS Ratio			
		**0.04**	**0.1**	**0.25**	**0.3**
airplane	ISTA-Net+ [28]	22.88/0.6401	28.83/0.8383	33.73/0.9029	35.04/0.9193
	AMP-Net [29]	26.69/0.8146	32.68/0.9258	39.08/0.9786	40.66/0.9852
	OPINE-Net+ [30]	27.18/0.8179	32.74/0.9220	38.87/0.9754	40.43/0.9819
	ours	**27.49/0.8289**	**32.89/0.9263**	**39.45/0.9776**	**40.84/0.9836**
buildings	ISTA-Net+ [28]	18.49/0.5271	24.13/0.7977	32.25/0.9459	33.89/0.9594
	AMP-Net [29]	23.03/0.7782	28.94/0.9213	36.06/0.9812	37.93/0.9873
	OPINE-Net+ [30]	23.19/0.7689	29.19/0.9206	35.87/0.9783	37.69/0.9846
	ours	**23.44/0.7797**	**29.37/0.9242**	**36.89/0.9816**	**38.42/0.9862**
dense residential	ISTA-Net+ [28]	19.43/0.5557	24.69/0.7896	31.82/0.9434	33.47/0.9599
	AMP-Net [29]	23.40/0.7487	28.49/0.9132	35.63/0.9800	37.56/0.9867
	OPINE-Net+ [30]	23.88/0.7667	29.11/0.9182	35.80/0.9793	37.62/0.9855
	ours	**24.15/0.7793**	**29.71/0.9276**	**36.69/0.9822**	**38.38/0.9874**
freeway	ISTA-Net+ [28]	21.29/0.5380	27.05/0.8132	33.21/0.9401	34.49/0.9533
	AMP-Net [29]	24.48/0.7296	29.54/0.9018	36.07/0.9757	37.67/0.9832
	OPINE-Net+ [30]	25.46/0.7640	30.64/0.9148	36.37/0.9742	37.86/0.9814
	ours	**25.89/0.7827**	**30.91/0.9177**	**36.99/0.9767**	**38.31/0.9824**
intersection	ISTA-Net+ [28]	20.50/0.5483	26.40/0.7763	33.12/0.9211	34.49/0.9381
	AMP-Net [29]	24.82/0.7433	29.84/0.8904	36.67/0.9706	38.42/0.9801
	OPINE-Net+ [30]	25.06/0.7496	30.43/0.8906	36.61/0.9671	38.29/0.9766
	ours	**25.20/0.7539**	**30.58/0.8919**	**37.42/0.9703**	**38.94/0.9788**
mobile home park	ISTA-Net+ [28]	17.37/0.4904	22.33/0.7338	29.75/0.9269	31.68/0.9480
	AMP-Net [29]	21.10/0.6985	25.94/0.8808	32.40/0.9683	34.11/0.9772
	OPINE-Net+ [30]	21.54/0.7213	26.53/0.8896	32.86/0.9674	34.40/0.9750
	ours	**21.94/0.7439**	**26.81/0.8954**	**33.66/0.9712**	**34.99/0.9777**
overpass	ISTA-Net+ [28]	22.87/0.5520	27.11/0.7481	34.19/0.9326	36.14/0.9550
	AMP-Net [29]	25.34/0.7140	29.69/0.8590	36.54/0.9695	38.36/0.9789
	OPINE-Net+ [30]	25.57/0.7182	30.69/0.8860	37.82/0.9739	39.09/0.9796
	ours	**26.07/0.7182**	**31.53/0.9069**	**38.54/0.9772**	**39.92/0.9826**
tennis court	ISTA-Net+ [28]	20.80/0.4438	23.71/0.6024	28.63/0.8407	30.29/0.8806
	AMP-Net [29]	23.68/0.5943	25.98/0.7494	29.22/0.8891	30.32/0.9124
	OPINE-Net+ [30]	23.71/0.5956	26.54/0.7763	30.83/0.9075	31.93/0.9243
	ours	**23.84/0.6075**	**27.19/0.8079**	**31.61/0.9176**	**32.43/0.9293**

**Table 4:** Average GPU runtime of six CS reconstruction algorithms for reconstructing 512 × 512 images at a sampling rate of 0.25.

Methods	AdapReconNet	CSNet+	ISTA-Net+	AMP-Net	OPINE-Net+	Ours
Time	0.0027 s	0.0007 s	0.0143 s	0.1270 s	0.0101 s	0.1950 s
#Para	1.15 M	1.17 M	0.34 M	0.58 M	1.10 M	2.23 M

**Table 5:** Average GPU runtime required to reconstruct images of 5 different sizes on MMU-Net.

Size	64 × 64	128 × 128	256 × 256	512 × 512	1024 ×1024
Time	0.0278 s	0.0350 s	0.0761 s	0.1950 s	0.7250 s

**Table 6:** Average PSNR and SSIM of the reconstructed images of the four CS reconstruction methods under the remote sensing image dataset with four sampling rates of 0.04, 0.1, 0.25, and 0.3. Bold indicates the best reconstruction performance, while underline represents the second-best reconstruction performance.

Dataset	Methods	Multi-Channel	Attention	0.1	0.25	0.3
Set11	GDM-(a)	×	×	29.79	34.81	36.04
	GDM-(b)	✓	×	29.90	35.01	36.17
	GDM-(c)	✓	✓	29.95	35.06	36.26
	GDM-(d)(ours)	✓	✓	**30.05**	**35.16**	**36.41**
UC Merced Land Use Dataset	GDM-(a)	×	×	29.48	35.62	37.16
	GDM-(b)	✓	×	29.60	35.90	37.30
	GDM-(c)	✓	✓	29.67	35.97	37.40
	GDM-(d)(ours)	✓	✓	**29.76**	**36.11**	**37.56**

**Table 7:** Average PSNR of reconstructed images for four network branching structures at three sampling rates (0.1, 0.25, and 0.3) on Set11 and the UC Merced Land Use Dataset, demonstrating the effectiveness of the multi-scale strategy. Bold indicates the best reconstruction performance, while underline represents the second-best reconstruction performance.

Dataset	Methods	0.1	0.25	0.3	0.25	0.3
Set11	Block-(1)	29.79	34.81	36.04	34.81	36.04
	Block-(2)	29.86	34.89	36.14	35.01	36.17
	Block-(3)	29.92	34.98	36.25	35.06	36.26
	Block-(4)	**29.98**	**35.10**	**36.35**	**35.16**	**36.41**
UC Merced Land Use Dataset	Block-(1)	29.48	35.62	37.16	35.62	37.16
	Block-(2)	29.56	35.72	37.15	35.90	37.30
	Block-(3)	29.62	35.84	37.27	35.97	37.40
	Block-(4)	**29.70**	**35.86**	**37.36**	**36.11**	**37.56**

## Data Availability

Data will be made available upon reasonable request.

## Abbreviations

The following abbreviations are used in this manuscript:

CS	Compressed Sensing
MMU-Net	Multi-channel and Multi-scale Unfolding
DNUN	Deep Non-unfolding Network
DUN	Deep Unfolding Network
CNN	convolutional neural network
AMP	Approximate Message Passing
ISTA	Iterative Shrinkage Thresholding Algorithm
ADMM	Alternate Direction Multiplier Method
AMGDM	Attention-based Multi-channel Gradient Descent Module
MPMM	Multi-scale Proximal Mapping Module
MB	Multi-scale Block
SS	Sampling Subnet
IS	Initialize Subnet
DRS	Deep Reconstruction Subnet
GDM	Gradient Descent Module
PMM	Proximal Mapping Module
PSNR	Peak Signal-to-Noise Ratio
SSIM	Structural similarity index Measure
